# Changes in Anxiety and Stress Among Pregnant Women During the COVID-19 Pandemic: Content Analysis of a Japanese Social Question-and-Answer Website

**DOI:** 10.2196/27733

**Published:** 2021-07-15

**Authors:** Ritsuko Shirabe, Tsuyoshi Okuhara, Rie Yokota, Hiroko Okada, Eiko Goto, Takahiro Kiuchi

**Affiliations:** 1 Department of Health Communication Graduate School of Medicine The University of Tokyo Tokyo Japan; 2 Department of Health Communication School of Public Health The University of Tokyo Tokyo Japan

**Keywords:** anxiety, content analysis, COVID-19, health communication, health information, mental health, pregnancy, social question-and-answer website, social support, stress

## Abstract

**Background:**

The changing pattern of anxiety and stress experienced by pregnant women during the COVID-19 pandemic is unknown.

**Objective:**

We aimed to examine the sources of anxiety and stress in pregnant women in Japan during the COVID-19 pandemic.

**Methods:**

We performed content analysis of 1000 questions posted on the largest social website in Japan (Yahoo! Chiebukuro) from January 1 to May 25, 2020 (end date of the national state of emergency). The Gwet AC1 coefficient was used to verify interrater reliability.

**Results:**

A total 12 categories were identified. Throughout the study period, anxiety related to going outdoors appeared most frequent, followed by anxiety regarding employment and infection among family and friends. Following the declaration of the state of national emergency at the peak of the infection, infection-related anxiety decreased, whereas anxiety about social support and mood disorders increased. Stress regarding relationships appeared frequent throughout the pandemic.

**Conclusions:**

The sources of anxiety and stress in pregnant women in Japan changed during the pandemic. Our results suggest the need for rapid communications in the early phase of a pandemic as well as long-term psychosocial support to provide optimal support to pregnant women in Japan. Health care professionals should understand the changing pattern of requirements among pregnant women.

## Introduction

During a pandemic, public health professionals have to communicate health information to vulnerable people [1]. Pregnant women are considered vulnerable because of the unknown risks to their health and to the fetus, pregnancy-related treatment restrictions, and restrictions on the number of prenatal hospital visits. In addition to specific infection prevention measures, pregnant women also require psychological care during a pandemic as excessive anxiety and stress adversely affect both maternal and infant health [2]. Many previous studies have reported a higher incidence of anxiety among pregnant women during the ongoing COVID-19 pandemic, with a prevalence rate of 30% for severe depressive and anxiety symptoms [3,4].

Effective risk communication campaigns require a “social constructionist approach,” which sees risk as being constructed through social and cultural processes [5-7]. In this concept, perceived risk may fluctuate through social processes and professionals have to understand what the stakeholders may ask and expect at each stage of a pandemic. Previous studies have reported that increased distress among pregnant women has been attributed to various factors, such as the risk of perinatal infection, unpreparedness for delivery, and altered support relationships [8,9]. However, little is known about changes in the sources of anxiety and stress over time.

To cope with isolation associated with enforced lockdown measures during the COVID-19 pandemic, many individuals began to use the internet to search for information and connect with others [10]. There are many social question-and-answer (Q&A) websites, where individuals may freely post questions to be answered by other community members. In the past, content analysis of questions posted to Q&A websites provided valuable insights into negative feelings and anxiety [11]. Social Q&A sites can be similarly useful for rapid investigation of anxiety and stress in dynamic situations such as the COVID-19 pandemic.

This study aimed to identify whether and how the sources of anxiety and stress in pregnant women in Japan changed during the COVID-19 pandemic, by carrying out content analysis of a social Q&A website, to inform preparations for timely essential support for this population in the ongoing and future pandemics.

## Methods

### Material Collection

The data were extracted from all questions posted in Japanese on Yahoo! Chiebukuro [12], the largest social Q&A website in Japan, from January 1 to May 25, 2020 (the end date of the national state of emergency). The questions were identified through a web-based search of the Japanese terms “corona AND ninnpu (pregnant woman),” “corona AND ninnshin (pregnancy),” “corona AND syussann (birth),” “corona AND bunnben (delivery),” and “corona AND osann (delivery).” We included only questions posted by pregnant women and excluded those posted by women with intrauterine fetal death, those unrelated to anxiety or stress about COVID-19, and duplicate questions. Because the data were publicly available, the requirement for informed consent was waived.

### Coding Procedure

The unit of coding was each question. Author RS read the text of all questions and then inductively assigned codes and categories to the extracted descriptions of anxiety and stress. When a question expressed multiple sources of distress, we coded it into all applying categories or codes. We enumerated the questions in each category or code. RS also tabulated the posting dates to correlate changes with weekly events in Japan.

Two independent coders (RS an RY) coded 20% of the questions, and interrater reliability was measured using the Gwet AC1 coefficient [13]. We used the coding carried out by RS for the analysis.

Coding procedures were conducted using Excel (version 2011, Microsoft Inc). Statistical analyses were performed using R for Windows (version 4.0.2, R Foundation for Statistical Computing).

## Results

### Material and Coding

**Figure 1:**
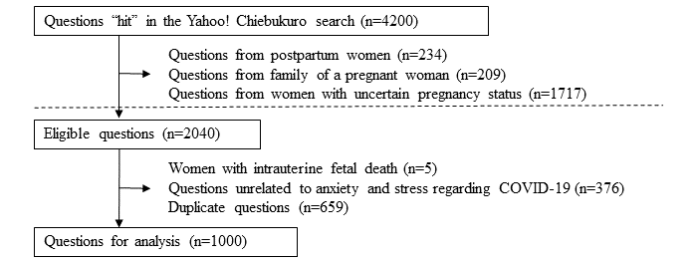
Flow chart for the selection of questions for analysis on Yahoo! Chiebukuro (the Japanese version of Yahoo! Answers), the largest social question-and-answer website in Japan.

A total of 4200 “hits” were obtained from the search terms, of which 2040 were questions posted by pregnant women. After excluding questions by women with intrauterine fetal death (n=5), unrelated questions (n=376), and duplicate questions (n=659), we retained 1000 questions for analysis (Figure 1). The questions had a median of 360 (IQR 228-546) Japanese characters and yielded 12 categories and 20 constituent codes (Table 1), which demonstrated strong interrater reliability (Gwet AC1=0.93, 95% CI 0.92-0.94). The total number of the codes was 1677 (median 1, IQR 1-2).

**Table 1:** Definitions of the assigned anxiety and stress categories and constituent codes.

Codes		Content	Example terms^a^ extracted from the questions
**Personal infection**			
	Risk of maternal infection	Anxiety about personal infection as a pregnant woman	Infection, coronavirus, maternal death, become severe, risk to pregnant women, immune weakness, and contraindications
	Pre-existing conditions or contacts with infected persons	Among nonpregnant women; anxiety about personal infection related to current symptoms, chronic conditions, or contact with a suspected infectious person	Fever, sore throat, asthma, and diabetes mellitus
	Infecting others	Anxiety about infecting others if asymptomatically infected	Family, other pregnant women, and canceled consultation
**Fetal well-being**			
	Adverse effects of infection	Anxiety about transmitting SARS-CoV-2 to the fetus or neonate	Baby, fetus, newborn, protect, mother-to-child transmission, abortion, stillbirth, disability, and sequelae
	Adverse effects of stress	Anxiety about adverse effects of maternal stress	Baby, fetus, newborn, and stress
**Going outdoors**			
	Daily life (eg, going to work)	Anxiety or stress about going outdoors for each purpose	Shopping, supermarket, workplace, and government offices
	Hospital visits (including prenatal check-ups)	Anxiety or stress about going outdoors for each purpose	Hospital, prenatal check-up, parents’ or mother’s class, and dentist
	Family events	Anxiety or stress about going outdoors for each purpose	Wedding, graduation ceremony, and funeral
	Social or leisure activities	Anxiety or stress about going outdoors for each purpose	Party, travel, beauty salon, and zoo
**Infection among family and friends**			
	Daily life (eg, going to work or school)	Anxiety or stress about contacts, including the behavior of those close to the person, such as partner, family members, and friends and workplace colleagues	Workplace, business trip, school, kindergarten, hospital, and daily shopping
	Family events	Anxiety or stress about contacts, including the behavior of those close to the person, such as partner, family members, and friends and workplace colleagues	Wedding, graduation ceremony, and funeral
	Undesirable outings or behaviors	Anxiety or stress about contacts, including the behavior of those close to the person, such as partner, family members, and friends and workplace colleagues	Dinner party, drinking party, travel, gambling, unwelcome visit, visit after delivery, not washing hands, and not wearing a mask
**Relationships**			
	Discord from risk behavior	Anxiety or stress and deteriorating relationships over others’ risk behaviors	Quarrel, get angry or annoyed, dislike, untrustworthy, divorce, and yelling at child
	Discord from each other factors (eg, spending more time together, estrangement, and stress)	Distress about relationships with partners, children, family, or friends, apart from risk behavior	Quarrel, get angry or annoyed, dislike, untrustworthy, divorce, and yelling at child
Mood disorders		Extreme feelings of sadness or depression	Cry, depressed, anxiety, finding it hard to live, wanting to die, abuse, symptoms of stress (eg, arrhythmia, stomach ache), and postponed or canceled ceremony
**Employment**			
	Financial insecurity	Anxiety about money	Salary, unpaid, allowance, retirement, unemployment,
	Treatment as pregnant woman at the workplace	Anxiety or stress about workplace practices or system, such as being forced to work or forced retirement	Forced to work, reduced working hours, maternity or child care leave, intends to leave, harassment, and revealing the pregnancy
Self-isolation		Anxiety or stress about self-isolation or staying at home	Stay at home, all day long, meal, exercise, and neighborhood noise
Daily necessities or hygiene products		Anxiety about daily necessities or hygiene	Lifeline, mask, thermometer, baby supplies, hoarding, and lack
Delivery facility		Anxiety or stress about the delivery facility (excluding prenatal visits and delivering alone)	Delivery facility, large hospital, returning to parental home around delivery (*Satogaeri*), transfer, waiting time, postponed check-ups, and cesarean section
**Society**			
	Policy	Anxiety or stress about social policies or systems	Government, municipality, system, emergency economic measures, and cash payment
	Actions or words of community members	Anxiety or stress about talk or actions of people in the community	Hoarding, blame, and public
**Social support**			
	Whether to accept support	Stress about accepting support	Self-restraint, parents, friends, homecoming, school, and kindergarten
	Lack of support or isolation	Anxiety about lack of support or isolation	Alone, no one to talk to, lonely, delivering alone, visit restrictions, father, refused consultation, and caring for a child alone

^a^Terms have been translated from Japanese into English in this publication.

### Distribution of Categories and Codes

**Figure 2:**
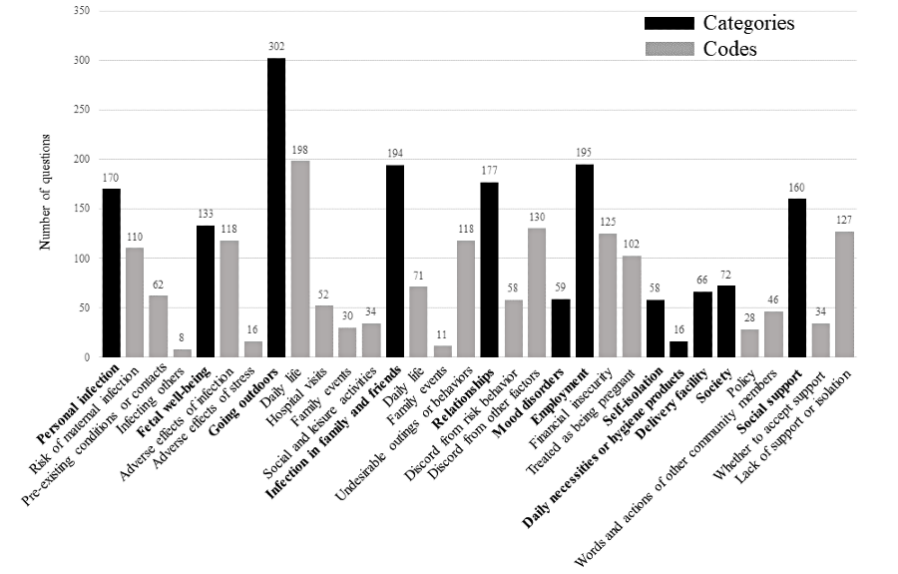
Frequency distribution of the anxiety and stress categories and codes described in the search questions. Some questions included multiple categories and codes.

Figure 2 shows the frequency distribution of the assigned categories and codes throughout the study period. The number for each category refers to the total number of applied questions and not the sum of the codes. Anxiety about going outdoors was most frequent, followed by anxiety related to work and anxiety related to infection among family and friends. Within the category “going outdoors,” anxiety or stress about infection in daily life (including going to work) was predominant, while in the category “infection in family and friends,” anxiety or stress related to undesirable activity was most frequent.

### Changes in the Frequency Distribution of Categories

**Figure 3:**
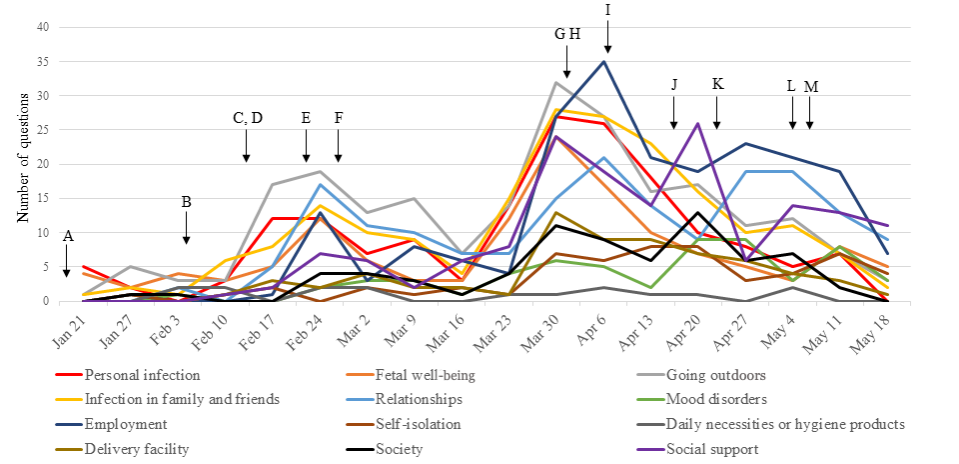
Weekly changes in the distribution of anxiety and stress categories and the chronology of key events among pregnant women in Japan. A: first COVID-19 case in Japan (January 16, 2020), B: report of a neonate infected with COVID-19 in China (February 5, 2020), C: first COVID-19 death in Japan (February 13, 2020), D: infection in a male physician; community infection suspected (February 13, 2020), E: official announcement of candidate drugs, including one contraindicated among pregnant women (February 22, 2020), F: nationwide school closures in Japan (February 27, 2020), G: report of a severe infection in a neonate in Japan (April 1, 2020), H: official request to economic and labor organizations for special considerations for pregnant workers (April 1, 2020), I: declaration of a state of emergency in 7 prefectures (April 7, 2020), J: declaration of a national state of emergency (April 16, 2020), K: reports of refusal among rural hospitals to accept transfers of pregnant women (April 23, 2020), L: extension of the national state of emergency (May 4, 2020), and M: amendment of labor guidelines that enable leave of absence from work among pregnant workers during the COVID-19 pandemic (May 7, 2020).

Figure 3 shows the weekly changes in the frequency distribution of the anxiety categories and　the chronology of key events among pregnant women in Japan. There were 3 peaks in the number of questions: the first peak occurred in the week of February 24, 2020, which coincided with the first wave of infection spreading from China; the second peak occurred in the week of April 6, 2020, which coincided with the sharp increase in infection with the second wave spreading from Europe and the United States; and the third peak occurred in the week of May 4, which coincided with the extended state of emergency. Infection-related anxiety (maternal infection, going outdoors, and infection among family and friends), and contributed to the first and the second peaks but declined during the third peak, during which anxiety about social support and mood disorders increased. Questions related to relationship stress were frequent throughout the study period.

## Discussion

### Principal Findings

During the COVID-19 pandemic, pregnant women in Japan expressed anxiety about infection and work as well as stress regarding relationships and social support. The sources of anxiety and stress changed over time; infection-related anxiety increased during the early phase of rapid pandemic growth, while anxiety about social support and mood disorders increased with the extending period of self-isolation.

### Anxiety and Stress in Pregnant Women

According to previous studies, many pregnant women were anxious about using public transportation and about infection among family members during the COVID-19 pandemic [14,15]. Pregnant women and new mothers were also uniquely impacted by stress related to reduced social and emotional support or family conflicts during the pandemic [9]. The anxiety and stress identified in this study is consistent with those reported previously. During the outbreak, the prevalence of severe depressive or anxiety symptoms among pregnant women increased with an increase in the number of cases and deaths [4]. Further, up to 30% of pregnant women reported experiencing anxiety symptoms even during the remission phase [16]. Given these findings, our study suggests the need for different types of care for pregnant women during a pandemic; rapid intervention is warranted during early stages of infection spread, whereas long-term support is needed even as the infection begins to wane.

At the beginning of the COVID-19 pandemic, risk communication campaigns seemed to fail owing to their reliance on a realist approach [6,7]. In this study, pregnant women frequently posted questions regarding their anxiety and stress related to maternal infection during the early spread of the pandemic. It has been reported that among expectant mothers, anxiety was heightened upon learning about infected new mothers and the lack of information from their physicians [17]. Pregnant women may perceive risk uniquely because the risk of affecting offspring is likely to be deemed greater [18]. Health care providers should use a variety of communication channels and added information resources to distribute audience-specific health messages [1]. Our results further suggest the need for specific messages that target family and friends—undesirable behaviors in the people surrounding the pregnant woman account for most of the anxiety about infection in family and friends as well as for relationship stress. Through a constructionist approach, there may be a need for different types of messages for partners and family members.

As the period of self-isolation became prolonged, pregnant women frequently posted about mood disorders and the lack of social support. A previous study reported that minimal contact with health care providers and the lack of routine nursing care during the outbreak contributes to social isolation [17]. Provision of perinatal care as part of infection prevention protocols requires ingenuity. Our results also suggest that health care providers should continue to evaluate psychological distress in pregnant women during this extended period.

### Implication in Practice and Future Studies

During a crisis, professionals must attempt to identify the needs of people in a timely manner because their needs may change with time or through social processes. In the expanding phase of a pandemic, health care providers should provide information regarding infection to pregnant women and their support network. With prolonging self-isolation, pregnant women also require psychosocial support. Evaluation of interventions including virtual perinatal care [19] will be needed in future.

### Limitations

This study has some limitations. Although our results do not differ from those of previous studies, the descriptions of anxiety and stress posted on the social Q&A site may have been biased. Although Yahoo! Chiebukuro is widely used (resolving approximately 75,000 questions per month), its use as the data source may have introduced a selection bias, given that the characteristics of the user population were unknown. The assigned codes may also have reflected an author bias. Despite these limitations, to our knowledge, this is the first study to show the changing pattern of anxiety and stress in pregnant women during the COVID-19 pandemic.

### Conclusions

Our findings show that pregnant women in Japan experienced anxiety and stress about infection in the early stages of the COVID-19 pandemic; however, over time, they increasingly experienced mood disorders and distress about the lack of social support. Professionals must understand these changing needs of vulnerable populations for effective communication during a crisis.

## Abbreviations

Q&A: question-and-answer
